# Emergency Surgical Treatment of a Large Pedunculated Subserosal Uterine Leiomyoma With Torsion: A Rare Cause of Acute Abdomen

**DOI:** 10.7759/cureus.52136

**Published:** 2024-01-11

**Authors:** Anna Thanasa, Efthymia Thanasa, Vasiliki Grapsidi, Ioannis Paraoulakis, Ioannis Thanasas

**Affiliations:** 1 Department of Health Sciences, Medical School, Aristotle University of Thessaloniki, Thessaloniki, GRC; 2 Department of Obstetrics and Gynecology, General Hospital of Trikala, Trikala, GRC

**Keywords:** pedunculated subserosal leiomyoma, torsion, abdominal pain, preoperative diagnosis, emergency surgical treatment, case report

## Abstract

Torsion of pedunculated subserosal leiomyoma of the uterus is a rare cause of acute abdomen. A 27-year-old female patient with twisted subserosal pedunculated uterine leiomyoma misdiagnosed as an adnexal mass underwent laparotomy. The patient came to the Emergency Department of General Hospital of Trikala with symptoms of acute abdomen. Primarily, clinical-laboratory examination, transabdominal ultrasound, raised the suspicion of a twisted adnexal mass. Intraoperatively, a twisted pedunculated subserosal leiomyoma of the uterus was identified and myomectomy was performed. Histological examination of the surgical specimen confirmed the diagnosis. On the third postoperative day, our patient was discharged. This paper underscores the rarity of torsion of the uterine pedunculated subserosal leiomyoma and emphasizes the necessity of urgent surgical intervention. The challenges of preoperative diagnosis are highlighted, especially when modern diagnostic imaging modalities are unavailable.

## Introduction

Leiomyomas are the most common benign neoplasms of the uterus, typically occurring during reproductive age [1]. In comparison to white women, where they are diagnosed in about 70%, leiomyomas are even more prevalent in women of African descent (>80%) [2]. Uterine fibroids are usually classified into intramural, submucosal and subserosal leiomyomas. A significant increase in the size of subserosal leiomyomas may lead to the development of pedunculated uterine mass [3]. Torsion of a subserosal pendunculated leiomyoma of uterus is rare. Due to the scarcity of large clinical studies, estimating the prevalence of uterine subserosal leiomyomas with torsion of the pedicle is difficult. Lai et al. retrospectively reviewed the medical records of patients with subserosal uterine leiomyomas who underwent surgery. The findings revealed that the incidence of torsion of a pedunculated subserosal uterine leiomyoma is less than 0.25%. Additionally, an increased risk of torsion in cases where the pedicle of the leiomyoma is thin and long [4].

This paper underscores the rarity of torsion of the uterine pedunculated subserosal leiomyoma and emphasizes the necessity of urgent surgical intervention. The challenges of preoperative diagnosis are highlighted, especially when modern diagnostic imaging modalities are unavailable.

## Case presentation

A 27-year-old age patient presented complaining of abdominal pain lasting approximately 12 hours. The pain was of sudden onset and was located especially in the right iliac fossa. In the last two hours, the intensity of the pain significantly increased, accompanied by episodes of vomiting. The patient did not complain of nausea, indigenous discomfort or unpleasant discomfort. No previous surgery was reported. Our patient was not sexually active. Her medical history included psychiatric disease, well-regulated with medication. The family history was unremarkable. Neither the mother nor the patient's sister reported benign or malignant diseases from the genital system.

The patient was afebrile and hemodynamically stable. During clinical examination, the patient exhibited tenderness. The examination was accompanied by signs of peritoneal irritation. Emergency blood tests revealed hematocrit 30.2%, hemoglobin 10.1gr/dl, platelets 245x103/ml, white blood cells 17.17x103/ml, neutral 87.9%, C reactive protein 17.06mg/dl. Biochemical analysis and urinalysis were in normal range. The pregnancy test was negative. Transabdominal ultrasound was not diagnostic due to the patient's increased body mass index (BMI = 32). The patient declined a transvaginal ultrasound, and a computed tomography scan was hindered by scanner malfunction. Magnetic resonance imaging was not available for the evaluation of abdominal pain in the emergency department of our hospital.

**Figure 1:**
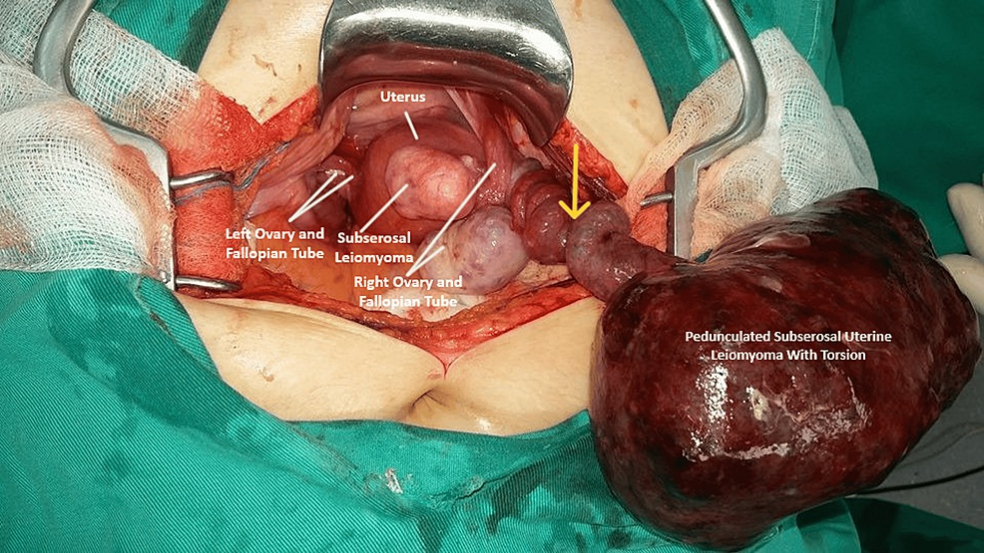
Intraoperative image depicting torsion of pedunculated subserosal uterine leiomyoma The presence of a long vascular pedicle (yellow arrow), combined with the large size of the tumor, significantly increases the risk of fibroid torsion

Based on the clinical-laboratory examination, with a lesser reliance on abdominal ultrasound, suspicion arose regarding the presence of a twisted adnexal mass. Consequently, the patient underwent emergency laparotomy by the gynecologists. Intraoperatively, upon entry into the peritoneal cavity, a twisted pedunculated solid mass originating from the uterus was identified, measuring approximately 15 cm in maximum diameter. The mass exhibited no adherence to adjacent tissues and showed signs of complete necrosis (Figure 1).

**Figure 2:**
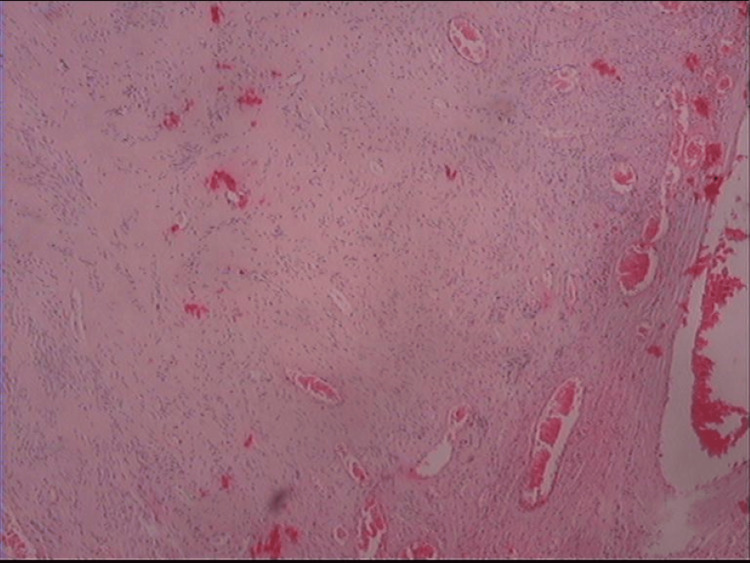
Histological image of twisted pedunculated subserosal uterine leiomyoma A subcellular leiomyomatous tumor without atypia and mitoses, with collagen deposition and with dilated congested vessels as a result of torsion is depicted

Surgical resection of the twisted uterine leiomyoma was performed. Histological examination of the surgical specimen confirmed the diagnosis (Figure 2).

Our patient, after a smooth postoperative course, was discharged from the Obstetrics and Gynecology Department of General Hospital of Trikala on the third postoperative day.

## Discussion

The clinical diagnosis of torsion of pedunculated subserosal uterine fibroids is challenging. Delayed diagnosis can result in ischemia and necrosis, leading to peritonitis and significant morbidity [5]. Generally, pedunculated subserosal uterine leiomyomas are usually asymptomatic or may be associated with varying degrees of pelvic compression symptoms. Acute abdominal pain is not common. However, the sudden onset of acute abdominal pain, as observed in our patient, is typically attributed to complete torsion of the vascular pedicle, followed by hemorrhagic necrosis of the leiomyoma [6]. Additionally, cystic degeneration of the tumor or infection represents other serious complications of pedunculated subserosal uterine leiomyomas which can cause an acute abdomen [7]. It's crucial to note that ovarian tumor torsion is the most common emergency gynecological condition and must necessarily be considered in the differential diagnosis of acute abdomen, along with torsion of pedunculated subserosal uterine fibroids [8].

Although the diagnosis of torsion of a pedunculated subserosal uterine fibroid is typically confirmed intraoperatively in most cases, modern imaging modalities can provide valuable preoperative information [9]. Doppler ultrasonography can raise suspicion of leiomyoma torsion by detecting reduced blood supply when the vascular pedicle is visible [10]. Additionally, contrast-enhanced computed tomography scan significantly contributes to the evaluation of acute abdominal pain caused by leiomyoma torsion and aid in excluding conditions related to this acute pain [5]. Magnetic resonance imaging, although not usually available for acute pain evaluation in the emergency department, contributes significantly to the diagnosis of twisted uterine leiomyoma by imaging the pedicle connecting the uterus to the necrotic leiomyoma [11]. In our patient, her refusal to undergo transvaginal ultrasound due to the absence of sexual intercourse, combined with the unavailability at that specific time of a computed tomography scanner (out of order due to malfunction) and a magnetic resonance imaging scanner in the emergency department, posed a significant diagnostic challenge. It is important to note that routine implementation of magnetic resonance imaging for the evaluation of abdominal pain in the emergency department is difficult. In our patient, the diagnosis of acute abdomen and the decision for surgery was based on the remarkable clinical symptoms and elevated inflammatory markers revealed by emergency blood tests.

Surgery is the primary treatment option for pedunculated subserosal uterine fibroids with torsion of the pedicle. Urgent or elective myomectomy via laparotomy or laparoscopy is the treatment of choice in young women who desire to preserve the uterus and achieve future pregnancy [12]. Total hysterectomy with bilateral adnexectomy is selected in older women [12]. Conservative management with uterine arteries embolization may be considered in the treatment of pedunculated subserosal uterine leiomyomas prior to torsion. In these cases it is thought to have a low risk of adverse events and to effectively reduce tumor volume [13].

## Conclusions

In conclusion, the presented case highlights the rarity and diagnostic challenges associated with torsion of pedunculated subserosal uterine leiomyoma, emphasizing the need for urgent surgical intervention in the setting of acute abdomen. While modern imaging methods can aid in preoperative diagnosis, the clinical presentation and elevated inflammatory markers played a crucial role in guiding timely surgical intervention, ultimately leading to a successful outcome for the patient.
